# Mass drug administration for the control and elimination of *Plasmodium vivax* malaria: an ecological study from Jiangsu province, China

**DOI:** 10.1186/1475-2875-12-383

**Published:** 2013-11-01

**Authors:** Michelle S Hsiang, Jimee Hwang, Amy R Tao, Yaobao Liu, Adam Bennett, George Dennis Shanks, Jun Cao, Stephen Patrick Kachur, Richard GA Feachem, Roly D Gosling, Qi Gao

**Affiliations:** 1 Global Health Group, University of California San Francisco (UCSF), San Francisco, USA; 2 Department of Pediatrics, UCSF Benioff Children’s Hospital, UCSF, San Francisco, CA 94143, USA; 3 Malaria Branch, Centers for Disease Control and Prevention, Atlanta, GA, USA; 4 School of Medicine, UCSF, San Francisco, USA; 5 Jiangsu Institute of Parasitic Diseases, Key Laboratory of Parasitic Disease Control and Prevention (Ministry of Health), Jiangsu Provincial Key Laboratory of Parasite Molecular Biology, Wuxi, China; 6 Department of Global Health Systems and Development, Tulane University School of Public Health and Tropical Medicine, New Orleans, LA, USA; 7 Australian Army Institute, Enoggera, Queensland, Australia

**Keywords:** Malaria elimination, Mass drug administration, Primaquine, *Plasmodium vivax*, China

## Abstract

**Background:**

Recent progress in malaria control has caused renewed interest in mass drug administration (MDA) as a potential elimination strategy but the evidence base is limited. China has extensive experience with MDA, but it is not well documented.

**Methods:**

An ecological study was conducted to describe the use of MDA for the control and elimination of *Plasmodium vivax* in Jiangsu Province and explore the association between MDA and malaria incidence. Two periods were focused on: 1973 to 1983 when malaria burden was high and MDA administered to highly endemic counties province-wide, and 2000 to 2009, when malaria burden was low and a focal approach was used in two counties. All available data about the strategies implemented, MDA coverage, co-interventions, incidence, and adverse events were collected and described. Joinpoint analysis was used to describe trends in incidence and the relationship between MDA coverage and incidence was explored in negative binomial regression models.

**Results:**

From 1973 to 1983, MDA with pyrimethamine and primaquine was used on a large scale, with up to 30 million people in target counties covered in a peak year (50% of the total population). Joinpoint analyses identified declines in annual incidence, -56.7% (95% CI -75.5 to -23.7%) from 1973–1976 and -12.4% (95% CI -24.7 to 2.0%) from 1976–1983. Population average negative binomial models identified a relationship between higher total population MDA coverage and lower monthly incidence from 1973–1976, IRR 0.98 (95% CI 0.97 to 1.00), while co-interventions, rainfall and GDP were not associated. From 2000–2009, incidence in two counties declined (annual change -43.7 to -14.0%) during a time when focal MDA using chloroquine and primaquine was targeted to villages and/or individuals residing near passively detected index cases (median 0.04% of total population). Although safety data were not collected systematically, there were rare reports of serious but non-fatal events.

**Conclusions:**

In Jiangsu Province, China, large-scale MDA was implemented and associated with declines in high *P. vivax* malaria transmission; a more recent focal approach may have contributed to interruption of transmission. MDA should be considered a potential key strategy for malaria control and elimination.

## Background

Mass drug administration (MDA) is a core strategy used for the control and elimination of many tropical diseases, including trachoma, lymphatic filariasis, schistosomiasis and onchocerciasis [1]. For malaria eradication in the 1950s and 60s, MDA was advocated by the World Health Organization (WHO) as a strategy in situations where more conventional control measures such as indoor residual spraying (IRS) with dichlorodiphenyltrichloroethane (DDT) had failed to end residual transmission. Defined as empiric administration of a therapeutic regimen to an entire population or well-defined sub-population at the same time, MDA campaigns worldwide utilized a variety of different drugs, regimens and dosing intervals. Most were associated with declines in incidence or parasite prevalence during the intervention but did not focus on species-specific impact [2] and were not designed to show interruption of transmission [3-5]. Due to concerns about efficacy, operational feasibility, sustainability, and emergence of drug resistance, MDA fell eventually out of favour.

Recent progress in malaria control has caused renewed enthusiasm and interest in MDA as a potential strategy for elimination and eradication [6,7]. MDA has also been considered as a strategy to contain the recent emergence of artemisinin resistance at the Cambodia-Thai and Thai-Myanmar borders [8,9]. However, recent evidence to guide MDA as a public health intervention for malaria remains sparse. Since the eradication era, studies of MDA have been limited [10-12] and large-scale use of drugs in communities has been focused on targeted therapies in specific populations such as in intermittent preventative therapy and seasonal malaria chemoprevention [13,14]. Furthermore, there has been less attention on MDA specifically for *Plasmodium vivax*, the species which becomes the ultimate challenge for most eliminating countries because it is more resilient than *Plasmodium falciparum* to standard interventions outside Africa [15]. *Plasmodium vivax* is able to persist in a dormant liver (hypnozoite) stage and relapse over long intervals of months to years. Primaquine, the only drug widely available for radical cure has variable efficacy and poor adherence due to the long treatment course required and concerns about potential life-threatening haemolysis in persons with underlying glucose-6-phosphate dehydrogenase (G6PD) deficiency [16]. An argument for MDA, *versus* screening and treatment, is that diagnosis is hampered by the lack of biomarkers for the liver stage, and limitations of microscopy or rapid diagnostic tests (RDTs) to detect blood stage infections which are characteristically of lower density than *P. falciparum*[17]. Furthermore, anopheline mosquito species that transmit *P. vivax* tend to be outdoor biting and resting making standard vector control measures such as bed nets and IRS less effective [18].

China has extensive experience with MDA, including for *P. vivax*, but this historical experience focusing specifically on MDA has not been well documented [3,4,19,20]. After the eradication era of the 1950s and 60s, falciparum malaria was eliminated from large parts of the country. But, the challenge of *P. vivax* persisted, particularly in the form of resurgences in central China occurring approximately every decade [21-27]. In the 1970s and 80s large-scale MDA was used to control these epidemics. In the 1990s and thereafter, a focal approach was used. The goal of this study was to document this MDA experience with *P. vivax*, focusing on Jiangsu Province for two time periods for which detailed records were available. The first period from 1973-1983 began with large *P. vivax* outbreaks which had been occurring since the Cultural Revolution (1965–68), during which time civil disturbances resulted in the abandonment of anti-malarial activities [21,22]. Mass drug administration was performed on a large scale involving millions of people. The second period focuses on 2000-2009, which began with an outbreak of *P. vivax* malaria in nearby Anhui Province and bordering areas of Jiangsu [28]. Focal MDA was performed on a smaller scale, targeted to close contacts, households and villages of index cases. The impact of MDA on incidence is also explored, acknowledging limitations of an ecological study design, which can show associations and generate hypotheses but cannot prove causality.

## Methods

### Study design

**Figure 1:**
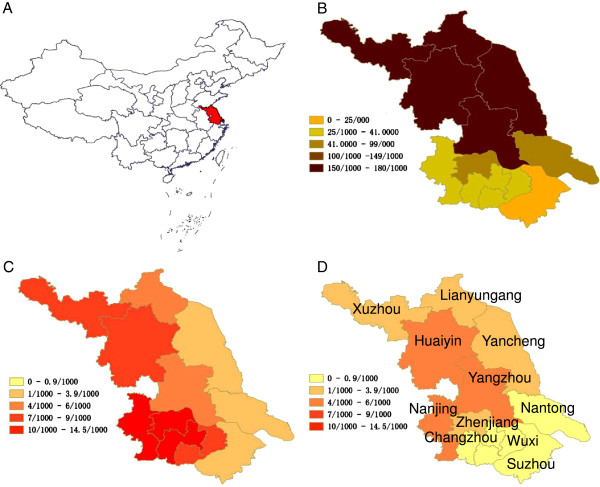
**Incidence maps of Jiangsu Province, 1973-1983. A)** Map of China showing location of Jiangsu Province, **B)** Prefecture level incidence in Jiangsu Province, 1973, **C)** Prefecture level incidence in Jiangsu Province, 1976, **D)** Prefecture level incidence in Jiangsu Province, 1983.

An ecological study was performed to describe the use of mass drug administration for control and elimination of *P. vivax* malaria in Jiangsu Province, China (Figure 1A), and to explore the impact on malaria incidence. While MDA had been used since the 1950s, two periods identified by the provincial malaria programme as having detailed records available were focused on: a) 1973 to 1983 with MDA to target counties in the province, and b) 2000 to 2009 with focal MDA to villages and close contacts of index cases in select counties during a *P. vivax* epidemic. As individual level data on malaria cases and compliance with MDA and other interventions were not available, population level figures were utilized.

### MDA methodology, 1973–1983

The goal of seasonal MDA with pyrimethamine and primaquine was to treat potential reservoirs of *P. vivax* blood and liver stage infection before the start of the malaria transmission season. *Plasmodium falciparum* had nearly been eliminated from Jiangsu Province by this time, accounting for fewer than 140 annual cases reported (Table 1).

**Table 1:** Jiangsu province, 1973-1983: annual malaria cases and intervention coverage

**Year**	**Total Population**	***Pf *cases**	***Pv *** **Incident cases**	***Pv *Incidence (/1,000)**	**MDA courses administered n (% total population)**	**Received MDA/Incident cases**	**Prophylactic courses administered**^**a**^** n (% total population)**	**Drug salts dispensed**^**b**^**n (% total population)**	**Bed nets n (% total population)**
1973	54,703,334	13	6,216,128	113.6	13,389,482 (24.5)	2	14,414,382 (3.3)	0 (0)	0 (0)
1974	54,252,430	4	3,341,110	61.6	17,746,954 (32.7)	5	23,926,883 (5.5)	0 (0)	0 (0)
1975	55,976,643	3	855,105	15.3	27,974,966 (50.0)	33	39,737,778 (8.9)	0 (0)	0 (0)
1976	56,630,708	137	359,371	6.3	27,329,972 (48.3)	76	44,185,317 (9.8)	237,376 (0.1)	0 (0)
1977	57,373,529	110	417,393	7.3	16,534,356 (28.8)	40	39,602,226 (8.6)	72,934 (0.0)	0 (0)
1978	58,025,577	13	640,434	11.0	10,591,797 (18.3)	17	38,866,265 (8.4)	466,594 (0.2)	42,744 (0.1)
1979	58,617,708	39	528,914	9.0	12,740,820 (21.7)	24	30,009,631 (6.4)	3,100,570 (1.3)	49,705 (0.1)
1980	58,921,850	135	377,166	6.4	11,787,687 (20.0)	31	25,034,512 (5.3)	4,156,676 (1.8)	84,425 (0.1)
1981	59,695,818	29	401,556	6.7	6,780,603 (11.4)	17	13,387,109 (2.8)	5,142,527 (2.2)	147,348 (0.2)
1982	60,466,585	67	252,832	4.2	5,503,181 (9.1)	22	9,533,912 (2.0)	5,840,737 (2.4)	142,066 (0.2)
1983	60,223,458	126	128,458	2.1	4,446,687 (7.4)	35	5,504,112 (1.1)	80,804 (0.0)	391,795 (0.7)

*Pf* Plasmodium falciparum, *Pv P. vivax.*

^a^Total amount of pyrimethamine prophylaxis given for the year. Administration was every two weeks June to October.

^b^Total amount of drug salts given for the year. Administration was monthly June to September.

**Figure 2:**
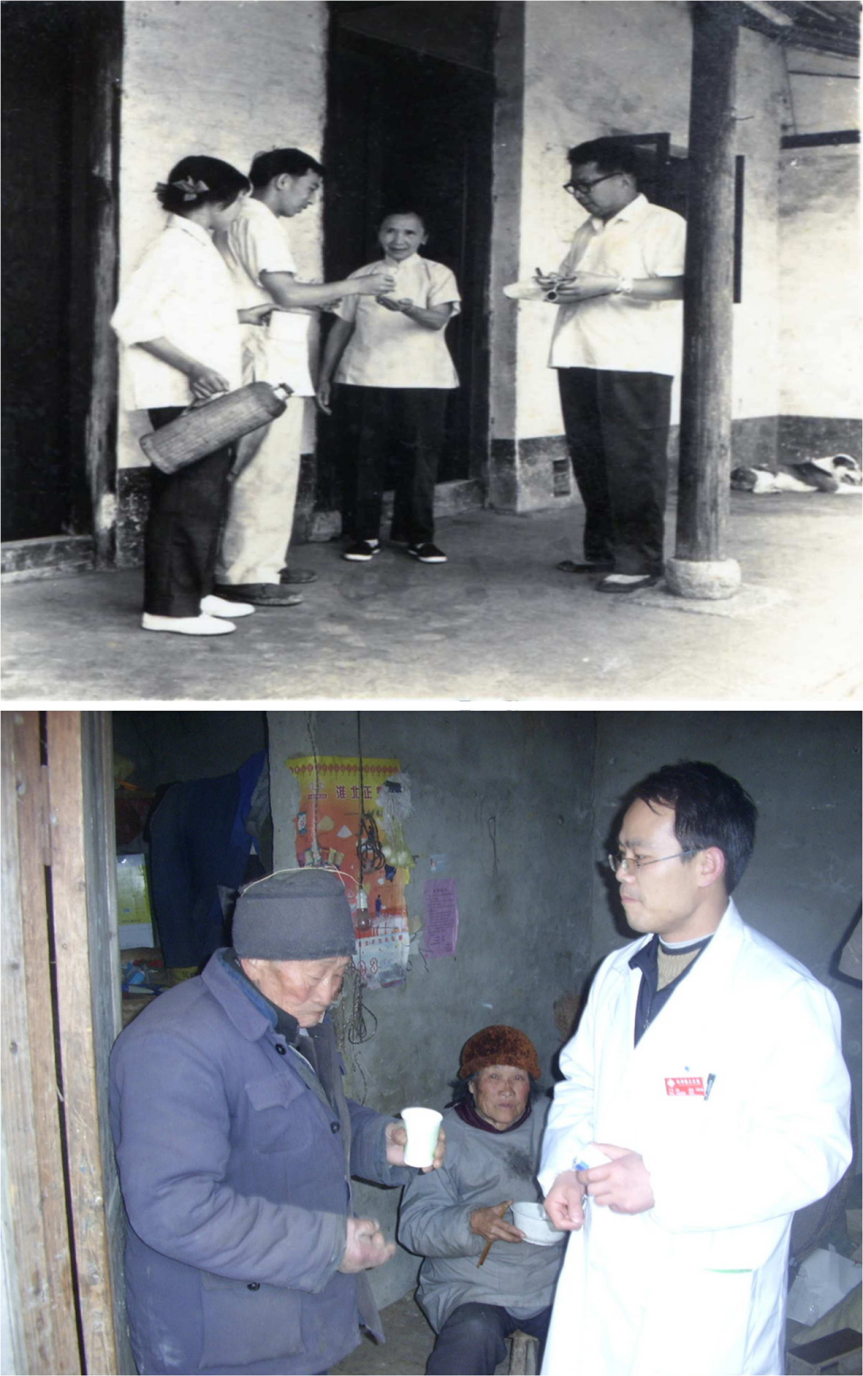
**Mass drug administration to villagers by directly observed therapy. A)** Xuzhou prefecture 1974, **B)** Sihong county 2009.

Villagers were notified prior to the annual campaign. Community health workers and local public health officers administered medications daily by directly observed therapy (Figure 2A). MDA was administered before or at the start of the malaria transmission season that typically begins in April. From 1973 to 1976, seasonal MDA using pyrimethamine and primaquine was directed at all rural counties, and from 1977 to 1983, a stratified approach using chloroquine and primaquine was implemented (Table 2).

**Table 2:** Stratification for seasonal mass drug administration

**Time period**	**Incidence in year prior**	**Target population**	**Daily drug regimen (adult dose)**
MDA, Jiangsu Province, 1973-1976			
1973-1976	n/a	All rural counties	pyrimethamine 50 mg × 2d + primaquine 30 mg × 4d
1977-1983	≥5%	Entire county	pyrimethamine 12.5 mg + primaquine 22.5 mg × 8d
	<5%	Index cases from past year with family and neighbors	
Focal MDA, Select counties, 2000-2009			
2000-2006	≥1%	Entire village	chloroquine 400 mg × 3d + primaquine 22.5 mg × 8d
	<1%	Index cases from past 2 y with family, IFAT positive individuals from past 2 y	
2007	≥1%	Entire village	chloroquine 400 mg × 3d + primaquine 22.5 mg × 8d
	<1% but >1 case	Index cases from past 2 y with family and neighbors	
	1 case	Index cases and IFAT positive individuals from past 2 y	primaquine 22.5 mg × 8d
2008	≥3%	Entire village	chloroquine 400 mg × 3d + primaquine 22.5 mg × 8d
	1-3%	Index cases from past year with family and neighbours	
	<1%	Index cases from past year with family, IFAT positive individuals from past year	primaquine 22.5 mg × 8d
2009	2-3 cases	Index cases from past year with family and neighbours	chloroquine 400 mg × 3d + primaquine 22.5 mg × 8d
	1 case	Index case from past year	primaquine 22.5 mg × 8d

IFAT indirect fluorescent antibody test, IFAT surveys as a measure of past exposure were performed in select townships. In Sihong, number of IFAT positive individuals treated was 24 in 2006, 13 in 2007, and six in 2008. Village refers to a natural village, the administrative level below an administrative village.

Drug regimens are recorded as adult dosages though age adjusted doses were given to children.

### MDA methodology, 2000–2009

From 2000–2009, focal “spring treatment” was administered in counties on the western border as response to a *P. vivax* epidemic due to larger outbreaks in nearby Anhui Province. Initially, entire natural villages (population ~20 persons, the division below an administrative village) were targeted. As incidence declined, more focal approaches were taken based on the provincial mandate (Table 2). Exclusion criteria included: age < one year, pregnancy, serious heart, liver or kidney disease, fever, and history of cyanosis, systemic bleeding, or dark coloured urine.

MDA was conducted by local hospital and health bureau workers in the homes of targeted individuals. Informed consent was conducted verbally and through a handout. Villagers were instructed to have food before taking the medication. The administration of drugs was by directly observed therapy (Figure 2B) and documented by collecting patient signatures. Medication was stopped if serious adverse effects such as dark urine, cyanosis, or haemolysis occurred. The goal was for 85% of individuals infected in the previous year to complete the full course and for 80% of other individuals (family, neighbours, etc.) to complete the full course. All medication administration and data collection were to be completed by the end of March of that year [29].

### Data collection

#### 1973–1983

Aggregate data from all 11 prefectures of Jiangsu Province were collected including monthly and annual incidence of reported malaria cases, annual number of people reached with MDA using pyrimethamine and primaquine prior to the malaria transmission season, annual prefecture population (based on annual census data), annual quantities of drug salts and other prophylactic medications administered, and vector control measures [30]. Protocols and reports on haemolysis or other adverse events relating to MDA were reviewed.

### 2000–2009

Aggregate data were gathered from two counties (the administrative level below prefecture) in the western part of Jiangsu where focal MDA was implemented and detailed records were available: Sihong in Suqian prefecture and Xuyi in Huaian prefecture (Suquian and Huaian previously combined and known as Huaiyin prefecture) [31,32]. The data included monthly and annual incidence, as well as number of subjects that were targeted and successfully completed a MDA course. Data on adverse events and coverage of vector control measures were only available from Sihong. Annual population was available for all townships (administrative level below county) within Sihong and 22 of 28 in Xuyi [33,34]. For missing data, populations were imputed based on average township population that year. Published literature as well as county and provincial records were reviewed for any data on haemolysis or other adverse effects relating to focal MDA.

For both time periods, gross domestic product (GDP) data from China were collected from the World Bank and deflated to year 2000 US$ to account for inflation. All monthly rainfall data were collected from the International Research Institute for Climate and Society [35]. For the 1978 to 1983 period, monthly rainfall data were calculated as the average from 14 weather stations throughout Jiangsu Province. For the 2000 to 2009 period, average monthly rainfall data were collected from two weather stations closest by coordinates to the selected counties.

### Data management and analysis

Data were extracted from paper records with support of a translator, and then double entered into Microsoft Excel (Microsoft Corp, Seattle, USA). Data were cleaned and analysed in R (Foundation for Statistical Computing, Vienna, Austria) and Stata 11.0 (Stata Corp, College Station, TX, USA).

Total MDA coverage was defined as per cent of the total population covered by MDA (total population coverage). This value did not take into account the fact that only high-risk or target villages and individuals received MDA. Therefore, target population coverage, or per cent of the target population covered by MDA for the 2000-2009 dataset (data not available for 1973-1983 dataset) was also presented. Also, as a functional measure that has relevance to operations and implementation, particularly for focal MDA where only villages or household members and neighbours are treated, the ratio of population receiving MDA to incident case was also calculated.

Given that incidence rate appeared to vary over the study time period, trends were analysed using joinpoint regression analysis, a statistical method that fits a series of joined straight lines to rates over time and chooses best-fitting points or joinpoints where the rate of increase or decrease changes significantly [36]. The grid search method was used to detect segments best describing the data. This method creates a “grid” of all possible locations for joinpoints and tests the error sum of squares at each one to find the best possible fit. Joinpoints were identified as years in which a significant change in trend was detected over the study period. Annual per cent change with 95% CI for each segment are reported to describe linear trends by period.

For the 1973-1983 Jiangsu Province dataset, the association between annual total population MDA coverage and incidence was estimated using population average negative binomial models. To control for background incidence trend as a potential confounder, an interaction term for time period was included based upon segments identified in the joinpoint analysis: early (1973-1976) *vs* later (1977-1983). A Poisson model was considered but not used as the negative binomial distribution provided a better fit for the data given the high amount of over-dispersion. The negative binomial analysis modelled monthly counts (monthly cases of *P. vivax*) as the outcome, taking into account prefecture population as an offset. Regression coefficients were computed as incidence rate ratios (IRRs). Total annual population MDA coverage was the primary exposure variable and other covariates controlled for including: annual chemoprophylaxis coverage, net coverage (defined as the number of nets distributed per population), rainfall lagged by one month, and deflated GDP. Drug salts were not included in the model due to non-significant effect and collinearity with chemoprophylaxis and bed nets. Generalized estimating equations with an autoregressive correlation structure were used to control for serial correlation within prefecture time series. Monthly and prefecture dummy terms were used to control for seasonality effects beyond rainfall and unobserved prefecture fixed effects, respectively. To control for potential spatial endogeneity between prefectures, the model included anomalies for intervention terms (computed as the difference between annual coverage and the local mean coverage over the study period for each prefecture). Rainfall and GDP values were standardized to represent one standard deviation (by subtracting the mean and dividing by the standard deviation).To evaluate the association between MDA and incidence during different time periods, a binary term for early (1973-1976) vs. later (1977-1983) years was interacted with the MDA term. Confidence intervals (CI) of 95% are presented based upon robust standard errors. Due to low total population coverage and incidence, the model was not applied to the 2000-2009 county datasets.

### Ethical issues

Human subjects’ approval was not indicated because the surveillance data utilised was existing, aggregate and without personal identifiers. The analysis protocol was reviewed by the Centers for Disease Control and Prevention IRB and received non-research determination.

## Results

### 1973–1983, MDA, Jiangsu province

#### MDA coverage

Total population coverage of seasonal MDA increased from 24.5% in 1973 to 50% in 1975, then gradually declined to 7.4% in 1983 (Table 1). At peak coverage in 1975, almost 30 million people were treated. The median number of individuals who received MDA for every incident case of *P. vivax* malaria the previous year was 24, ranging from 2 in 1973 to 76 in 1976.

### Other interventions

In almost all prefectures, intermittent chemoprophylaxis was used during the transmission season from June to October. The regimen consisted of either pyrimethamine 50 mg every two weeks or chloroquine 300 mg every ten days. Per cent population coverage of chemoprophylaxis increased from 3.3% in 1973 to 9.8% in 1976, then declined to 1.1% by 1983. At the height of the programme, up to 44 million cumulative doses were administered a year. From 1976 to 1983, a few prefectures also distributed salt fortified with pyrimethamine. From June to September, each person received a monthly average of 400 g salt containing 157.5 mg pyrimethamine [37]. Population coverage was highest at 2.4% in 1982. Bed nets were utilized beginning in 1978, however population level coverage was very low. Indoor residual spraying was not part of the provincial malaria strategy.

Treatment for clinical malaria cases varied by county. The most commonly used regimen was chloroquine on days 1-3 (1.2 g total) with primaquine on days 1-4 (22.5 mg daily). One month later patients were treated with primaquine for another four days (22.5 mg daily) for a total primaquine dose of 180 mg. Another regimen used was chloroquine on days 1-3 (1.2 g total) with primaquine on days 1-8 (22.5 mg daily, 180 mg total). From 1973 to 1977, a five-day regimen was also used in some counties, chloroquine on days 1-3 (1.2 g total) with primaquine on days 1-5 (22.5 mg daily, 112.5 mg total). Subjects who were infected in the previous year were also individually targeted for seasonal MDA.

### MDA and incidence

**Figure 3:**
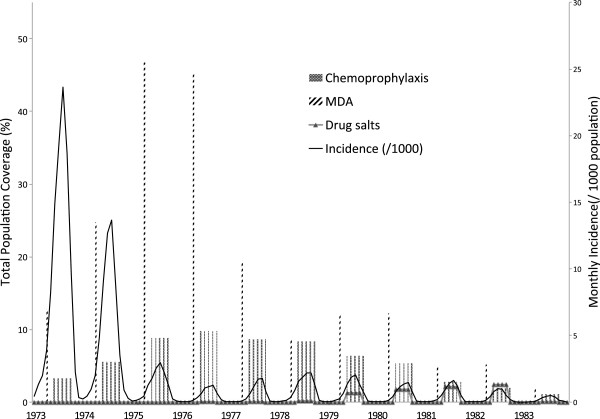
Monthly incidence and total population of mass drug administration, chemoprophylaxis and drug salts, Jiangsu Province, 1973-1983.

In the setting of large scale MDA, the incidence of *P. vivax* in Jiangsu Province declined from 113.6 in 1973 to 2.1 per 1,000 population in 1983 (Table 1, Figures 1B- 1D, 3). Joinpoint analysis on the aggregate provincial dataset identified two distinct periods: 1973-1976 when annual per cent change was -56.7% (95% CI -75.5 to -23.7%) and 1976-1983 when annual per cent change was -12.4% (95% CI -24.7 to 2.0%). At the prefecture level, no more than two distinct periods were identified, with joinpoint breaks occurring from 1976 to 1978. In most prefectures, annual per cent changes in incidence were negative (Table 3).

**Table 3:** Joinpoint analysis for malaria incidence trends in Jiangsu province

**MDA 1973−1983**	**Segments**	**Annual per cent change (95% CI)**
Province−wide	1973–1976*	−56.7 (−75.5 to −23.7)
	1976–1983	−12.4 (−24.7 to 2.0)
Prefectures		
Changzhou	1973–1983*	−33.8 (−36.7 to −30.8)
Huaiyin	1973–1983*	−19.9 (−33.0 to −4.2)
Lianyungang	1973–1983*	−24.3 (−38.2 to −7.3)
Nanjing	1973–1978	9.5 (−35.2 to 85.1)
	1978–1983*	−19.7 (−28.6 to −9.7)
Nantong	1973–1977*	−65.5 (−75.8 to −50.9)
	1977–1983	−6.1 (−22.3 to 13.5)
Suzhou	1973–1976	−8.6 (−40.2 to 39.8)
	1976–1983*	−35.7 (−42.6 to −28.0)
Wuxi	1973–1983*	−40.2 (−43.1 to −37.2)
Xuzhou	1973–1983*	−24.2 (−38.8 to −11.9)
Yancheng	1973–1976*	−71.6 (−84.1 to −49.3)
	1976–1983*	−18.6 (−30.2 to −4.8)
Yangzhou	1973–1976*	−67.6 (−82.2 to −41.1)
	1976–1983	−1.4 (−15.9 to 15.8)
Zhenjiang	1973–1977*	−44.2 (−56.2 to −28.9)
	1977–1983*	−28.1 (−36.8 to −18.1)
Focal MDA 2000−2009		
County		
Sihong	2000–2009*	−27.0 (−35.6 to −17.2)
Xuyi	2000–2002*	−43.7 (−65.7 to −7.8)
	2002–2009*	−14.0 (−24.8 to −1.7)

*statistically significant.

The population average negative binomial model confirmed that time period significantly impacted the relationship between total population MDA coverage and incidence: monthly incidence was significantly lower in the later period compared with the earlier period (IRR = 0.19; 95% CI 0.11 to 0.33), after controlling for potential confounding factors including chemoprophylaxis, bed nets, rainfall, and GDP (Table 4). Between 1973 and 1976, higher total population MDA coverage was associated with significantly lower monthly incidence (IRR = 0.98; 95% CI 0.97 to 1.00) after controlling for confounders, but in the later period, it was associated with higher incidence (IRR = 1.05; 95% CI 1.03 to 1.06). Other potential confounders were not significantly associated with monthly incidence.

**Table 4:** Coefficient estimates of population average negative binomial models predicting monthly cases

**Parameter**	**Incidence rate ratio (IRR) and 95% CI (n = 11 prefectures)**	
(1977-1983) *vs* (1973-1976)^a^	0.19^b^	(0.11 to 0.33)
Annual MDA: (1973-1976)^a^	0.98^c^	(0.97 to 1.00)
Annual MDA: (1977-1983)^a^	1.05^b^	(1.03 to 1.06)
Annual chemoprophylaxis	0.98	(0.96 to 1.00)
Bed nets per person	1.03	(0.94 to 1.13)
Rainfall (lagged 1 month)	1.02	(0.98 to 1.06)
GDP	0.95	(0.87 to 1.04)
n (prefecture-months)	1452	

Models include month dummies to control for seasonality and prefecture dummies to control for unobserved fixed effects (coefficient estimates not shown).

^a^adjusted analyses.

^b^p < 0.001.

^c^p < 0.05.

### Safety

Adverse events from MDA were not systematically monitored as part of the programme, but five reports documenting G6PD deficiency-related severe adverse events from primaquine were identified (Table 5). Two case series were recorded in the setting of large-scale campaigns, one from 1975 and one from 1976. There were two cross-sectional studies, one among family members of patients with severe G6PD deficiency and one among high-risk groups. Additionally, there was a case report from 1979.

**Table 5:** Summary of acute haemolysis reports, Jiangsu province

**Study design**	**Methods**	**Findings**
Case series [38]	Setting: 1975, Jinhu county, Huaiyin prefecture, 254,910 residents given primaquine (22.5 mg daily × 8 days) and pyrimethamine (50 mg daily × 2 days), Incidence of haemolysis: 3.5/100,000	Baseline characteristics of affected patients: 8 of 9 were male, mean age 14.4 (range 5 to 38), two brothers with history of favism.
	Methods: clinical course described in nine patients with acute haemolysis	Haemolysis on day 2 or 3 after cumulative dose of 45 mg in 2 adults and after 15-30 mg in children (ages 5 to 15)
		Symptoms: haematuria, weakness, fever, appetite loss, abdominal pain/discomfort, dizziness and headache, bruising, epistaxis
		Patients recovered with drug discontinuation, transfusions, and supportive care
Case series [39]	Setting: 1976, Linhe county, Nanjing prefecture, 444,589 residents given primaquine (30 mg daily × 4 days) and pyrimethamine (50 mg daily × 2 days), Incidence of haemolysis: 9.3/100,000	Baseline characteristics of affected patients: 32 of 40 were male, age range 4 to 62, 65% were <15 y, 20% with history of favism, 15% with family history of haemolysis
	Methods: collected clinical data in 40 patients with acute haemolysis (more detailed data from 18 hospitalized patients), methaemoglobin reductive testing in 80 family members (immediate and spouse) and 29 healthy controls	Haemolysis in 18 hospitalized patients: 56% onset 1-2 days after medication. Haemoglobin levels (g/dL): 3-5: 12 patients, 5.2-6.5: 7 patients, 7.5: 1 patient (unclear if repeated measured included)
		Symptoms: jaundice (18), fever (17), loss of appetite (14), weakness (12), dizziness (11), haematuria (9), dark coloured urine (8), abdominal pain (7), cyanosis (7), and headache (7)
		Deficiency by methaemoglobin testing: 65% of patients tested one month after haemolytic event, 28/50 (56%) female family including 12 of 29 mothers tested and 11 of 17 mothers of male patients, 6/30 (20%) male family (age range of male or female family members: 6-70 y), 0% in adult controls (53.8% male); deficiencies were medium except severe in 2 patients and 2 family members
		Household clustering rare (1 household with 3 cases, 2 households with 2 cases)
Cross-sectional survey [40]	Setting: as above Methods; methaemoglobin reductive testing in family members of 3 patients who had haemolysis after primaquine, 94/141 family members agreed to participate	Deficiency by methaemoglobin testing detected in 59.6%. Test results by sex (males n = 54, females n = 40): any deficiency: 51.9% in males *vs* 70.0% in females, serious deficiency: 51.9% in males *vs* 17.5% in females
Cross-sectional survey [41]	Setting: as above but in 1977 following mass drug administration Methods: methaemoglobin reductive testing in 1515 subjects from 4 comparison groups Comparison groups: 1) village with high incidence of haemolysis, 2) village with past *P. falciparum* cases, 3) village with Hui minority group, 4) patients with schistosomiasis associated liver disease or chronic hepatitis. All ≥5 years of age	Prevalence of deficiency: group 1: 5.7% (60/1051, 19 severe), group 2: 3/289 1.0% (1 severe), group 3: 0.7% (1/143), group 4 0% (0/32)
		Prevalence of deficiency by sex: males 4.9% vs females 3.8% (n=689 males and 801 females). Prevalence of deficiency in males by age: 5-9y 10.5% (n = 58), 10-14y 5.8% (n = 220), 15-18y 3.5% (n = 114), 19-30y 2.3% (n = 131), 31-60y 5.2% (n = 153), >60y 0.0% (n = 13). Prevalence of deficiency in females by age: 5-9y 9.7% (n = 62), 10-14y 3.5% (n = 170), 15-18y 2.5% (n = 120), 19-30y 2.5% (n = 303), 31-60y 4.5% (n = 224), >60y 0.0% (n = 22)
Case report [42]	Setting: 1979, Xinyi county, Xuzhou prefecture Methods: clinical course described	15 y male diagnosed with vivax malaria based on clinical symptoms of fever, sweats, headache. Developed fever and malaise after 2 days of chloroquine (900 mg) and primaquine (37.5 mg), next day had dark urine, jaundice, nausea, vomiting, fever, hepatomegaly, haematocrit 35%. Admitted and recovered with discontinuation of primaquine, transfusions, and supportive care.

Methaemoglobin reductive testing, severe 0-30%, medium 3175%, normal >75%.

In the two case series, drugs were administered to 254,910 residents in Jinhu county and 44,589 residents of Liuhe county. The incidence of acute haemolysis was 3.5 and 9.3 per 100,000 population, respectively. Symptoms were typical for acute haemolytic reaction. Among the 40 patients from Liuhe county, G6PD deficiency testing was performed using the methaemoglobin reductive test, which had a sensitivity of 65% for detection of patients with acute haemolysis. In both studies, there were no deaths and all patients recovered with drug discontinuation and supportive care.

In one of the cross-sectional studies, methaemoglobin testing was performed in 94 family members of three patients who suffered from haemolysis after primaquine and 59.6% tested positive. In another study, methaemoglobin testing was performed in groups hypothesized to have higher prevalence of G6PD deficiency. The prevalence of a deficiency was 5.7% in a village with a high incidence of acute haemolysis *versus* 0.7-1.0% in the other groups (a village with past *P. falciparum* cases and a village with members of the Hui minority group) and 0% in a control group of patients with liver disease. The higher prevalence in the village with a high incidence of acute haemolysis was thought to be due to a genetic predisposition in this community.

### 2000–2009, focal MDA, Sihong and Xuyi counties

#### Focal MDA coverage

**Figure 4:**
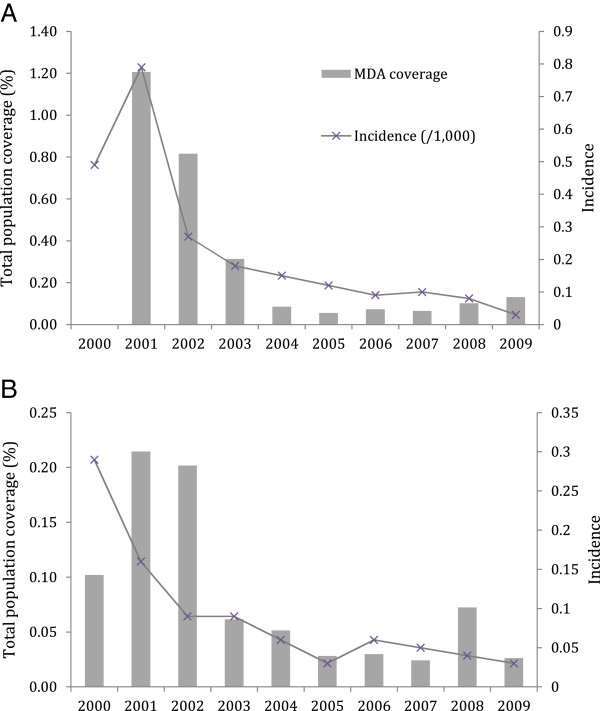
**Incidence with total population coverage of focal mass drug administration, 2000-2009. A)** Sihong county, **B)** Xuyi county.

Utilizing the stratified strategy, total population coverage was low. In 2001, it was 1.21% in Sihong and 0.21% in Xuyi, then dropped to very low levels by 2003 (Figure 4). On the other hand, target population coverage was generally high. In Sihong, median 91.6% (range 59.5 to 99.2%) completed treatment and in Xuyi, median 92.0% (range 76.5 to 98.3%) completed treatment. The median number of individuals who completed MDA for each incident case of *P. vivax* malaria was 16 (range 5 to 39) in Sihong and 10 (range 4 to 22) in Xuyi (Table 6).

**Table 6:** Sihong and Xuyi counties, 2000-2009: annual malaria cases and mass drug administration coverage

**Year**	**Total population**	**Incident cases**	**Incidence (/1000)**	**MDA courses completed**	**Total population coverage (%)**	**Target population coverage (%)**	**Completed MDA/Incident case**
Sihong							
2000	979107	477	0.49	0	n/a	n/a	n/a
2001	999522	788	0.79	12056	1.21	89.6	15
2002	1021073	273	0.27	8330	0.82	88.3	31
2003	1027329	188	0.18	3220	0.31	74.7	17
2004	979866	145	0.15	836	0.09	90.6	6
2005	979866	120	0.12	542	0.06	59.5	5
2006	1002408	93	0.09	730	0.07	92.6	8
2007	1010050	100	0.10	657	0.07	94.5	7
2008	1011239	83	0.08	1032	0.10	94.7	12
2009	1014450	34	0.03	1331	0.13	98.2	39
Xuyi							
2000	898854	263	0.29	917	0.10	98.3	4
2001	898854	144	0.16	1928	0.21	76.5	14
2002	898854	84	0.09	1814	0.20	90.4	22
2003	898854	79	0.09	554	0.06	89.5	7.
2004	898854	58	0.06	463	0.05	96.9	8
2005	883533	25	0.03	249	0.03	85.0	10
2006	883533	54	0.06	264	0.03	94.0	5
2007	883533	42	0.05	213	0.02	92.6	5
2008	883533	35	0.04	640	0.07	91.3	18
2009	883533	25	0.03	231	0.03	98.3	9

### Other interventions

Unlike in the 1973-1983 time period, other prophylactic drugs were not utilized. In Sihong county, there was some use of vector control measures. Insecticide-treated bed nets covered 0 to 3% of the population between 2000 and 2004, 10% in 2005, and 4 to 8% between 2006 and 2009. Indoor residual spraying was performed in 2007 and 2009 in select townships. Coverage was recorded as 109.5 kg sprayed over 47,706 sq m in 2007 and 231 kg sprayed over 109,125 sq m in 2009. No information was available on proportion of structures sprayed. There was no recorded use of vector control measures in Xuyi. During this time period, clinical cases of *P. vivax* presenting to health facilities were treated with chloroquine for three days (1.2 mg total dose) and primaquine for eight days (180 mg total dose).

### Focal MDA and incidence

Although the peak incidences were low at baseline, 0.79 and 0.29 per 1,000 population in Sihong and Xuyi, respectively, incidence declined further in the setting of focal MDA (Figure 4). Sihong and Xuyi counties saw declines the year after focal MDA was started. Low incidences were maintained even as focal MDA coverage declined. Joinpoint analysis identified two distinct periods in Xuyi and one in Sihong (Table 3). In Xuyi, the break correlated with a change in total population coverage of focal MDA (Figure 4). Annual per cent changes were negative and statistically significant in all segments. Temporally, other factors did not appear to contribute to decreasing incidence. Gross domestic product increased over the study period but the rate was steady. Rainfall was highest in 2000, 2005 and 2007 (Additional file 1: Figures S1b and S2b). Vector control measures were used in Sihong, but the periods of highest coverage do not correspond with declines in incidence.

### Safety

In Sihong, the number of subjects with adverse reactions was recorded in 2003 and 2007. Adverse reactions were few, occurring in five subjects (2.1/1,000) in 2003 and two (4.3/1,000) in 2007. Symptoms included haemolysis with primaquine, dizziness, gastrointestinal upset, nausea, and vomiting with chloroquine. Safety data were not available from Xuyi.

## Discussion

MDA is increasingly being considered in the battle against malaria yet few descriptions of its use for *P. vivax* exist in the published literature. This ecological study describes the methods and rationale behind the largest malaria MDA implementation experience documented to date. The potential impact of seasonal MDA on the incidence of vivax malaria in Jiangsu Province, China was explored. After controlling for potential confounders including economic growth, weather, and co-interventions, it was found that large-scale MDA from 1973-1976 was associated with declines in *P. vivax* malaria transmission. In the 2000-2009 data from two counties, joinpoint analyses showed significant declines in incidence that temporally appear related to focal MDA and not other factors. Safety data were not collected systematically but there were rare reports of serious but non-fatal events. More recent surveillance data indicate that the declines in malaria have been sustained. In 2011, 20 of China’s 31 provinces reported no locally acquired malaria cases. In 2012, Jiangsu was one of six additional provinces to report no locally acquired cases [43]. Thus, the large-scale approach was associated with declines in high levels of transmission and the more recent focal approach may have contributed to interruption of transmission.

With the renewed interest and commitment to malaria elimination, there is growing interest in MDA using primaquine because it is the only drug available to treat the hypnozoite stage of *P. vivax*, as well as mature gametocytes of *P. falciparum*[44]. Primaquine has been widely used for MDA, particularly during the eradication era, but detailed documentation on its use specifically for *P. vivax* has been sparse, there are no controlled studies, and few have demonstrated sustained impact [2,3,45-51]. This study had similar limitations in that it was an ecological study and not able to prove impact given the lack of a control. There is also the possibility of reverse causation. From 1977-1983, the model showed a positive association between MDA and incidence, but this is likely because total population coverage decreased as incidence declined during this distinct period. Also, in the later time period, total population coverage was lower and underestimated the true target population coverage to a greater degree. Other potential limitations include confounding and information bias. However, attempts were made to control for economic growth, weather and co-interventions. While it is possible that local officials reported inaccurate coverage and incidence figures, findings were consistent across several groups (prefecture and counties) and over a long time period.

Despite the limitations of an ecological study, the design enabled exploration of the impact of a population level intervention on a population level outcome, which is of obvious public health importance and should stimulate further scientific endeavours, both for intervention studies and the development of new drugs and diagnostics for use in MDA. This paper is among the few to systematically document the MDA experience from China, where the malaria experience is rich but poorly documented due to language barriers. It provides a rare focus on *P. vivax*, the most large-scale MDA experience documented (peak coverage reaching 30 million people), and is the first to describe China’s recent use of focal MDA.

The optimal timing, frequency, drug(s), target population, and target population coverage in MDA are not clear [8,52]. In Jiangsu, the timing (before the transmission season) and drugs (antirelapse +/- blood schizonticide) were selected to decrease the reservoir, rather than to produce an immediate impact on disease burden as the target population was largely asymptomatic. An eight-day course of primaquine was chosen based on efficacy seen in clinical trials and concern that a longer treatment course would be operationally challenging to administer via directly observed therapy and less acceptable to the population [53]. Also, although the treatment course was shorter than the standard 14-day course recommended by WHO, the daily dose was higher, and courses were repeated annually.

The target population was adapted to a changing epidemiology. During the higher burden period from 1973-1976, drugs were administered to entire high-risk counties and as transmission declined, a stratified approach was taken to target villages and households of passively identified index cases (assuming local transmission at the home residence) and eventually single, high-risk individuals (e g, subjects positive by indirect fluorescent antibody test (IFAT) testing). The stratified approach was undertaken as a way to decrease workload, but more importantly, to minimize risk and maximize acceptability in communities that no longer saw malaria as a major threat. Target population coverage data were not available for the 1973-1983 programme. However from 2000-2009, it was generally above 85% in Sihong and Xuyi. These goal coverage levels are similar to estimated necessary coverage for *P. falciparum* elimination [54]. To help guide MDA operations for *P. vivax*, future field and modelling work should aim to determine the minimal coverage required to achieve an objective (control or elimination).

As an additional measure of coverage, the ratio of population receiving MDA to incident case was calculated. Although this measure has not been used in prior MDA reports, it is presented as a functional measure that has relevance to operations and implementation, particularly for targeted MDA where only villages or household members and neighbours are treated. From 1973-1983, when MDA was administered to entire counties, the range was 2 to 76, with the ratio increasing as incidence declined. With the focal approach used from 2000-2009, the range was 4 to 39. Operationally, lower numbers correspond roughly to the size of a household of an index case, while larger numbers correspond with the involvement of neighboring households. This ratio could be considered similar to the concept of number needed to treat (NNT) and future work is needed to better define the optimal ratio.

A major challenge of primaquine use for MDA is the risk of haemolysis in patients with underlying G6PD deficiency. In symptomatic cases, testing for G6PD deficiency is not recommended prior to administration of a single dose to target *P. falciparum* gametocytes but it is recommended for a 7- to 14-day regimen for radical cure of *P. vivax*[55] and there are no guidelines for use in MDA of asymptomatic individuals. The reported prevalence of G6PD deficiency among Han, the predominant ethic group in Jiangsu is low, <5% among males [56]. In one of the case series study, the reported incidence of haemolysis from mass primaquine administration was 9.3 per 100,000 population [39]. The authors note that the incidence was higher than expected and this may have been because the daily primaquine dose of 30 mg was too high (subsequent campaigns used 22.5 mg) or because the timing of that year’s MDA campaign was later than previous years and coincided with peak seasonal fava bean consumption. The most comparable MDA to those reported here was conducted in 300,000 US soldiers returning from Korea on troopships in 1952-4 who received 210 mg of primaquine over two weeks. The severe haemolytic reaction rate was estimated at four per 100,000 in a population that would have been expected to have a greater prevalence of G6PD-deficient individuals than in China [57]. No irreversible adverse events occurred in the Jiangsu reports, but the potential risks suggest that in other settings with similar rates of G6PD deficiency, close monitoring, which would be possible with daily direct observed therapy, could be considered. In addition services for urgent blood transfusion should be available for the few cases that do haemolyse to very low levels of haemoglobin. Testing for G6PD deficiency might be focused on patients with a personal or family history of favism or haemolysis (Table 5) or performed on a more wide scale once a point of care G6PD deficiency test becomes available.

## Conclusions

There is growing interest for the use of MDA to control and eliminate malaria but the literature on use specifically for *P. vivax* is limited. While this study was retrospective and mainly descriptive, it adds to the evidence base for seasonal MDA. It presents a little documented experience from China, where seasonal MDA was practiced (first on a large scale and later in a focal fashion) and likely contributed to declines in vivax malaria transmission and rare reported adverse events. Ongoing research is needed to develop a safer and more effective drug, but there are likely settings where MDA using primaquine could be applied now with a logical and safe operational design and evaluated through a phased approach. MDA should be considered as a potential strategy to hasten and achieve malaria elimination, and not remain something of only historical interest.

## Abbreviations

CI: Confidence interval; G6PD: Glucose-6-phosphate dehydrogenase; GDP: Gross domestic product; IFAT: Indirect fluorescent antibody test; IRR: Incidence rate ratio; IRS: Indoor residual spraying; MDA: Mass drug administration; RDT: Rapid diagnostic test; UCSF: University of California, San Francisco; WHO: World health organization.

## Competing interests

The authors declare that they have no competing interests.

## Authors’ contributions

MSH, QG, RGAF, and GDS conceived the study. MSH, QG and JH designed the study. ART, YL and MSH collected the data. AB, JH, and MSH analysed the data. RG, SPK and CJ contributed to the interpretation of the data. MSH, ART, JH, and AB drafted the paper. All authors reviewed early drafts and contributed to the final paper. All authors read and approved the final manuscript.

## Supplementary Material

Additional file 1 Economic indicators and monthly rainfall. Click here for file

## References

[B1] Hotez PJ Mass drug administration and integrated control for the world’s high-prevalence neglected tropical diseases Clin Pharmacol Ther 2009 85 659 664 10.1038/clpt.2009.16 19322166

[B2] Onori E Experience with mass drug administration as a supplementary attack measure in areas of vivax malaria Bull World Health Organ 1972 47 543 548 4573215 PMC2480830

[B3] von Seidlein L Greenwood BM Mass administrations of antimalarial drugs Trends Parasitol 2003 19 452 460 10.1016/j.pt.2003.08.003 14519583

[B4] Poirot E Hwang J Kachur SP Slutsker L Skarbinski J Mass drug administration for malaria Cochrane Database Syst Rev 2010 CD008846 DOI: 008810.001002/14651858.CD14008846 10.1002/14651858.CD008846.pub2 PMC4468927 24318836

[B5] Greenwood B The use of anti-malarial drugs to prevent malaria in the population of malaria-endemic areas Am J Trop Med Hyg 2004 70 1 7 14971690

[B6] The malERA Consultative Group on Drugs A research agenda for malaria eradication: drugs PLoS Med 2011 8 e1000402 21311580 10.1371/journal.pmed.1000402 PMC3026688

[B7] Cotter C Sturrock HJW Hsiang MS Liu J Phillips AA Hwang J Smith-Gueye C Fullman N Gosling RD Feachem RGA The changing epidemiology of malaria elimination: new strategies for new challenges Lancet 2013 382 900 911 10.1016/S0140-6736(13)60310-4 23594387 PMC10583787

[B8] Maude RJ Socheat D Nguon C Saroth P Dara P Li G Song J Yeung S Dondorp AM Day NP White NJ White LJ Optimising strategies for *Plasmodium falciparum* malaria elimination in Cambodia: primaquine, mass drug administration and artemisinin resistance PLoS One 2012 7 e37166 10.1371/journal.pone.0037166 22662135 PMC3360685

[B9] World Health Organization Consideration of mass drug administration for the containment of artemisinin-resistant malaria in the Greater Mekong subregion, Report of a consensus meeting, 27-28 September 2010 2011 Geneva, Switzerland: World Health Organization

[B10] Kaneko A Taleo G Kalkoa M Yamar S Kobayakawa T Bjorkman A Malaria eradication on islands Lancet 2000 356 1560 1564 10.1016/S0140-6736(00)03127-5 11075770

[B11] Song J Socheat D Tan B Dara P Deng C Sokunthea S Seila S Ou F Jian H Li G Rapid and effective malaria control in Cambodia through mass administration of artemisinin-piperaquine Malar J 2010 9 57 10.1186/1475-2875-9-57 20175930 PMC2837675

[B12] Von Seidlein L Walraven G Milligan PJ Alexander N Manneh F Deen JL Coleman R Jawara M Lindsay SW Drakeley C De Martin S Olliaro P Bennett S Schim van der Loeff M Okunoye K Targett GA McAdam KP Doherty JF Greenwood BM Pinder M The effect of mass administration of sulfadoxine-pyrimethamine combined with artesunate on malaria incidence: a double-blind, community-randomized, placebo-controlled trial in The Gambia Trans R Soc Trop Med Hyg 2003 97 217 225 10.1016/S0035-9203(03)90125-8 14584381

[B13] Bardaji A Bassat Q Alonso PL Menendez C Intermittent preventive treatment of malaria in pregnant women and infants: making best use of the available evidence Expert Opin Pharmacother 2012 13 1719 1736 10.1517/14656566.2012.703651 22775553

[B14] Meremikwu MM Donegan S Sinclair D Esu E Oringanje C Intermittent preventive treatment for malaria in children living in areas with seasonal transmission Cochrane Database Syst Rev 2012 2 CD003756 10.1002/14651858.CD003756.pub4 PMC6532713 22336792

[B15] Feachem RG Phillips AA Hwang J Cotter C Wielgosz B Greenwood BM Sabot O Rodriguez MH Abeyasinghe RR Ghebreyesus TA Snow RW Shrinking the malaria map: progress and prospects Lancet 2010 376 1566 1578 10.1016/S0140-6736(10)61270-6 21035842 PMC3044848

[B16] John GK Douglas NM von Seidlein L Nosten F Baird KJ White NJ Price RN Primaquine radical cure of *Plasmodium vivax*: a critical review of the literature Malar J 2012 11 280 10.1186/1475-2875-11-280 22900786 PMC3489597

[B17] Douglas NM John GK von Seidlein L Anstey NM Price RN Chemotherapeutic strategies for reducing transmission of Plasmodium vivax malaria Advances in parasitology 2012 80 271 300 23199490 10.1016/B978-0-12-397900-1.00005-0

[B18] Sattabongkot J Tsuboi T Zollner GE Sirichaisinthop J Cui L *Plasmodium vivax* transmission: chances for control? Trends Parasitol 2004 20 192 198 10.1016/j.pt.2004.02.001 15099559

[B19] Dapeng L Leyuan S Xili L Xiance Y A successful control programme for falciparum malaria in Xinyang, China Trans R Soc Trop Med Hyg 1996 90 100 102 10.1016/S0035-9203(96)90099-1 8761560

[B20] Chen W Wu K Lin M Tang L Gu Z Wang S Lan C Lan X Li H Huang M Chen X Sheng H [A pilot study on malaria control by using a new strategy of combining strengthening infection source treatment and health education in mountainous areas of Hainan province](in Chinese) Zhongguo Ji Sheng Chong Xue Yu Ji Sheng Chong Bing Za Zhi 1999 17 1 4 12563805

[B21] Tang LH Qian HL Xu SH Malaria and its control in the People’s Republic of China Southeast Asian J Trop Med Public Health 1991 22 467 476 1820632

[B22] Tang LH Progress in malaria control in China Chin Med J 2000 113 89 92 11775219

[B23] Huang F Zhou S Zhang S Li W Zhang H Monitoring resistance of Plasmdium vivax: point mutations in dihydrofolate reductase gene in isolates from Central China Parasit Vectors 2011 4 80 10.1186/1756-3305-4-80 21586132 PMC3108914

[B24] Xu BL Su YP Shang LY Zhang HW Malaria control in Henan Province, People’s Republic of China Am J Trop Med Hyg 2006 74 564 567 16606984

[B25] Huang GQ Yuan FY Jin XL Zhao CL Su YP Shen YZ To analyze epidemic situation and control of malaria in Jiangsu, Shandong, Henan, Anhui, and Hubei province Chin J Vector Biol Control 2007 18 398 401

[B26] Sleigh AC Liu XL Jackson S Li P Shang LY Resurgence of vivax malaria in Henan Province, China Bull World Health Organ 1998 76 265 270 9744246 PMC2305715

[B27] Zhou SS Wang Y Tang LH [Malaria situation in the People’s Republic of China in 2006](in Chinese) Zhongguo Ji Sheng Chong Xue Yu Ji Sheng Chong Bing Za Zhi 2007 25 439 441 18441886

[B28] Zhang W Wang L Fang L Ma J Xu Y Jiang J Hui F Wang J Liang S Yang H Cao W Spatial analysis of malaria in Anhui province, China Malar J 2008 7 206 10.1186/1475-2875-7-206 18847489 PMC2572066

[B29] Sihong County Centers for Disease Control and Prevention Malaria anti-relapse therapy guidelines 2008 Sihong, Jiangsu, China: Sihong County Centers for Disease Control and Prevention

[B30] Jiangsu Institute of Parasitic Diseases Jiangsu Province Malaria Records 1973-1983 1984 Wuxi, Jiangsu, China: Jiangsu Institute of Parasitic Diseases

[B31] Xuyi County Centers for Disease Control and Prevention Xuyi County Malaria Reports 2000-2009 2010 Xuyi, Jiangsu, China: Xuyi County Center for Disease Control and Prevention

[B32] Sihong County Centers for Disease Control and Prevention Sihong County Malaria Reports 2000-2009 2010 Sihong, Jiangsu, China: Sihong County Centers for Disease Control and Prevention

[B33] Sihong County Centers for Disease Control and Prevention Sihong Statistical yearbooks 2000-2009 2010 Sihong, Jiangsu, China: Sihong County Bureau of Statistics

[B34] Xuyi County Centers for Disease Control and Prevention Xuyi Statistical yearbooks 2000-2009 2010 Xuyi, Jiangsu, China: Xuyi County Bureau of Statistics

[B35] International Research Institute for Climate and Society http://iridl.ldeo.columbia.edu

[B36] Stracci F Canosa A Minelli L Petrinelli AM Cassetti T Romagnoli C La Rosa F Cancer mortality trends in the Umbria region of Italy 1978-2004: a joinpoint regression analysis BMC Cancer 2007 7 10 10.1186/1471-2407-7-10 17227578 PMC1781946

[B37] Jiangsu Institute of Parasitic Diseases Jiangsu Eight Main Counties, Malaria Control Report 1984 Wuxi, Jiangsu, China: Jiangsu Institute of Parasitic Diseases

[B38] Jiangsu Institute of Parasitic Diseases Primaquine adverse reaction: 9 case reports, Jiangsu Province Schistosomiasis Prevention Research Center and Liuhe County Health and Immunization Station, in: Selected Malaria Records from 1974-1983 1984 Wuxi, Jiangsu, China: Jiangsu Institute of Parasitic Diseases 122

[B39] Jiangsu Institute of Parasitic Diseases Primaquine causing hemolysis, 40 cases report, Jiangsu Province Schistosomiasis Prevention Research Center and Liuhe County Health and Immunization Station, in: Selected Malaria Records from 1974-1983 1984 Wuxi, Jiangsu, China: Jiangsu Institute of Parasitic Diseases 104 107

[B40] Jiangsu Institute of Parasitic Diseases Survey of family members: 3 cases, Jiangsu Province Schistosomiasis Prevention Research Center and Liuhe County Health and Immunization Station, in: Selected Malaria Records from 1974-1983 1984 Wuxi, Jiangsu, China: Jiangsu Institute of Parasitic Diseases 116

[B41] Bian S Li C Zhang X Huang B Wang Y Zhao L Jiangsu Province Liuhe Region Hereditary RBC G6PD Defect Preliminary Investigation Report, Jiangsu Province Schistosomiasis Prevention Research Center and Liuhe County Health and Immunization Station, in: Selected Malaria Records from 1974-1983 1984 Wuxi, Jiangsu, China: Jiangsu Institute of Parasitic Diseases 110 115

[B42] Jiangsu Institute of Parasitic Diseases Primaquine adverse event: 1 case report, Jiangsu Province Schistosomiasis Prevention Research Center and Liuhe County Health and Immunization Station, in: Selected Malaria Records from 1974-1983 1984 Wuxi, Jiangsu, China: Jiangsu Institute of Parasitic Diseases 121

[B43] Yin JH Yang MN Zhou SS Wang Y Feng J Xia ZG Changing Malaria Transmission and Implications in China towards National Malaria Elimination Programme between 2010 and 2012 PLoS One 2013 8 e74228 10.1371/journal.pone.0074228 24040210 PMC3767829

[B44] White NJ Primaquine to prevent transmission of falciparum malaria Lancet Infect Dis 2013 13 175 181 10.1016/S1473-3099(12)70198-6 23182932

[B45] Shanks GD Control and elimination of *Plasmodium vivax* Adv Parasitol 2012 80 301 341 23199491 10.1016/B978-0-12-397900-1.00006-2

[B46] Garfield RM Vermund SH Changes in malaria incidence after mass drug administration in Nicaragua Lancet 1983 2 500 503 6136655 10.1016/s0140-6736(83)90523-8

[B47] World Health Organization Interregional workshop on the control of vivax malaria in East Asia, Shanghai, China, 2003 2004 Regional Office for the Western Pacific: World Health Organization

[B48] WHO Inter-regional meeting on vivax malaria in Asia 2009 Regional Office for South-East Asia: World Health Organization

[B49] Simeons ATW Follow-up of a mass treatment with injectable atebrin Ind Med Gaz 1938 73 713 715 PMC5219286 29014263

[B50] Caceres JL Malaria before and after massive radical cure in Sucre state, Venezuela Bol Malar Salud Ambi 2008 48 83 90

[B51] Gabaldon A Guerrero L An attempt to eradicate malaria by the weekly administration of pyrimethamine in areas of out-of-doors transmission in Venezuela Am J Trop Med Hyg 1959 8 433 439 13670370 10.4269/ajtmh.1959.8.433

[B52] Greenwood BM Control to elimination: implications for malaria research Trends Parasitol 2008 24 449 454 10.1016/j.pt.2008.07.002 18760671

[B53] Ho C Studies on malaria in new China Chin Med J 1965 84 491 497 5865019

[B54] Okell LC Griffin JT Kleinschmidt I Hollingsworth TD Churcher TS White MJ Bousema T Drakeley CJ Ghani AC The potential contribution of mass treatment to the control of *Plasmodium falciparum* malaria PLoS One 2011 6 e20179 10.1371/journal.pone.0020179 21629651 PMC3101232

[B55] White NJ Qiao LG Qi G Luzzatto L Rationale for recommending a lower dose of primaquine as a *Plasmodium falciparum* gametocytocide in populations where G6PD deficiency is common Malar J 2012 11 418 10.1186/1475-2875-11-418 23237606 PMC3546849

[B56] Jiang W Yu G Liu P Geng Q Chen L Lin Q Ren X Ye W He Y Guo Y Duan S Wen J Li H Qi Y Jiang C Zheng Y Liu C Si E Zhang Q Tian Q Du C Structure and function of glucose-6-phosphate dehydrogenase-deficient variants in Chinese population Hum Genet 2006 119 463 478 10.1007/s00439-005-0126-5 16607506

[B57] Alving AS Clinical treatment of malaria US Army Medical Science Publication No 4 1954 22 210 218

