# Supplementary material for: Mass drug administration for the control and elimination of Plasmodium vivax malaria: an ecological study from Jiangsu province, China
Source: Malar J. 2013 Nov 1;12:383. doi: 10.1186/1475-2875-12-383 (PMC3842644; doi:10.1186/1475-2875-12-383)

Supplemental

Figure S1. Economic indicators over the study period in each of the study sites, A. China deflated GDP 1973-1983, B. China deflated GDP 2000-2009

A.

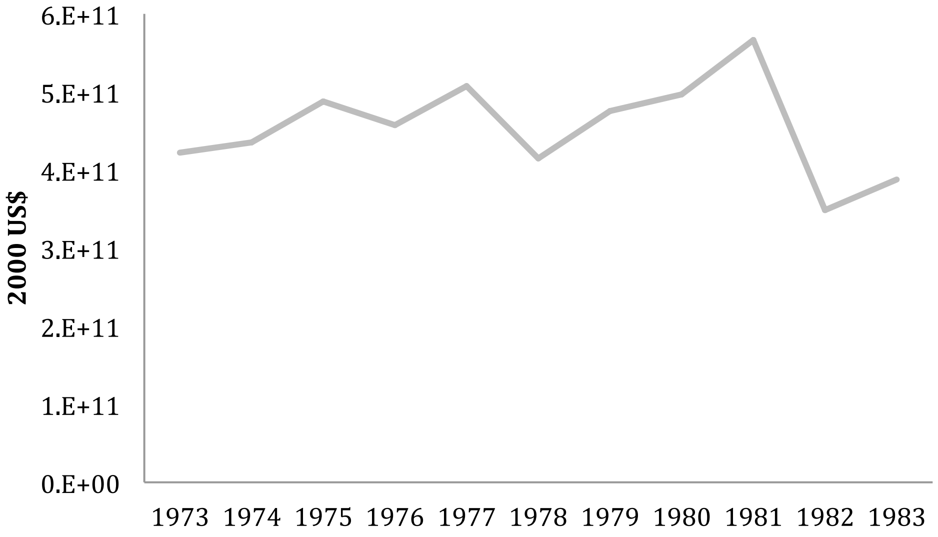

B.

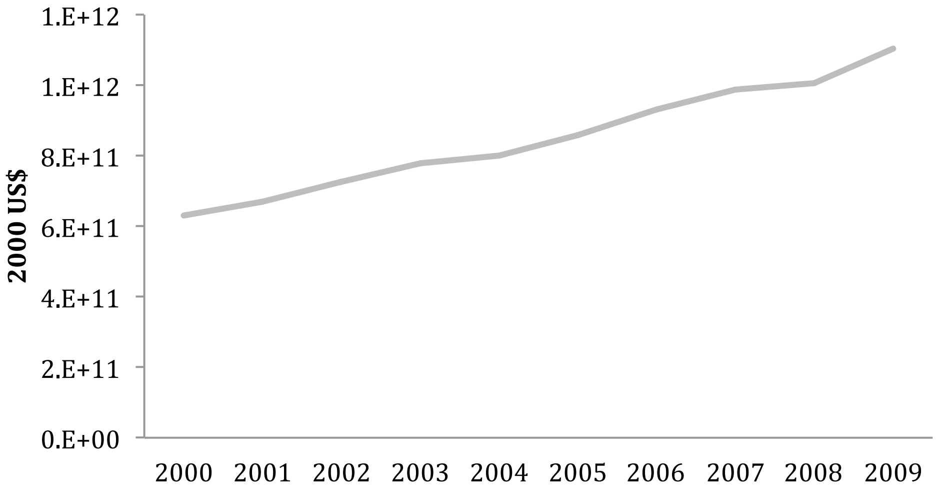

Figure S2. Monthy rainfall, A. 1973-1983, Aggregate data from 14 weather stations throughout Jiangsu Province, B. 2000-2009, Data from weather station closest to Sihong, Xuyi, and Suining counties

A.

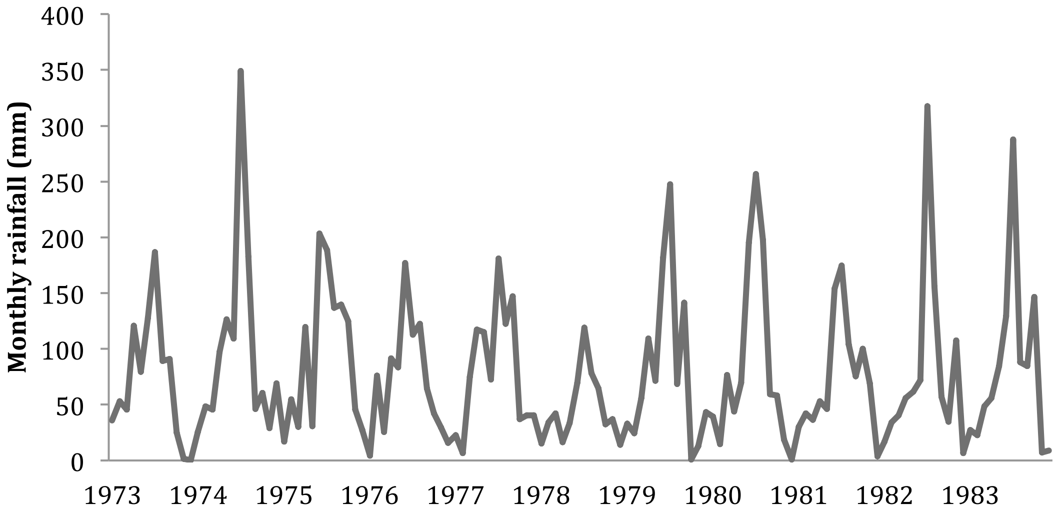

B.

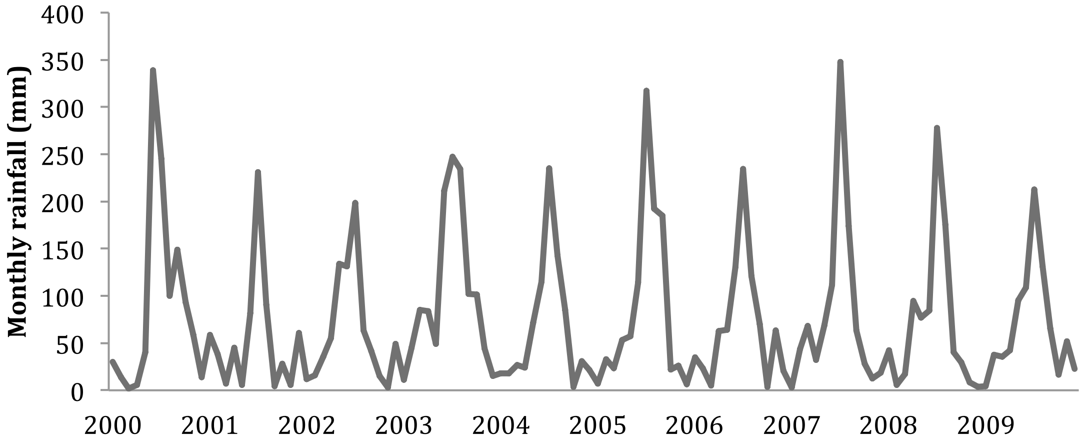

Supplement: Additional file 1 — Economic indicators and monthly rainfall. [file 1475-2875-12-383-S1.pdf]
